# Parvovirus B19 IgG-Defined Prior Exposure and Its Association with Anemia in Maintenance Hemodialysis Patients: A Cross-Sectional Comparative Study

**DOI:** 10.3390/jcm15124461

**Published:** 2026-06-09

**Authors:** Metin Özsoy, Salih Cesur, Mehmet Emin Demir, Feyza Bayrakdar Çağlayan, Murat Duranay, Uğur Hatipoğlu, Ramazan Öztürk, Simge Bardak Demir, Altan Aksoy

**Affiliations:** 1 Department of Infectious Diseases and Clinical Microbiology, Ankara Training and Research Hospital, Health Sciences University, 06230 Ankara, Türkiye; mozsoy@ada.net.tr (M.Ö.); scesur89@yahoo.com (S.C.); 2 Department of Nephrology, School of Medicine, Atılım University, 06830 Ankara, Türkiye; 3 Department of Nephrology, Yüksek İhtisas University, 06330 Ankara, Türkiye; feyzabayraktars@hotmail.com; 4 Department of Nephrology, Ankara Training and Research Hospital, Health Sciences University, 06230 Ankara, Türkiye; duranaymurat@hotmail.com (M.D.); ramazanozturk1977@yahoo.com (R.Ö.); 5 Department of Hematology and Apheresis Unit, Ankara Oncology Training and Research Hospital, Health Sciences University, 06200 Ankara, Türkiye; ugurugur2192@gmail.com; 6 Department of Nephrology, Yenimahalle Education and Research Hospital, Yıldırım Beyazıt University, 06370 Ankara, Türkiye; bardaksimge@gmail.com; 7 Department of Medical Microbiology, Ankara Training and Research Hospital, Health Sciences University, 06800 Ankara, Türkiye; aksoy.altan@hotmail.com

**Keywords:** Parvovirus B19, hemodialysis, anemia, seroprevalence, erythropoiesis-stimulating agents

## Abstract

**Background:** Parvovirus B19 (B19V) has a well-established tropism for erythroid progenitor cells and is a recognized cause of anemia in immunocompromised individuals. Patients with end-stage renal disease (ESRD) receiving maintenance hemodialysis are predisposed to anemia due to multiple mechanisms and are frequently exposed to healthcare settings, raising concern that prior B19V infection may contribute to anemia severity or resistance to erythropoiesis-stimulating agents (ESAs). However, data regarding the clinical relevance of B19V seroprevalence in hemodialysis patients remain limited. **Methods:** We conducted a single-center, observational cross-sectional study including 131 adult patients on maintenance hemodialysis and 50 healthy controls. Parvovirus B19 IgG serostatus was assessed by enzyme-linked immunosorbent assay (ELISA) and used exclusively as a marker of prior (past) exposure rather than active infection; our aim was to determine whether IgG-defined prior exposure leaves a measurable long-term imprint on erythropoiesis. None of the participants had clinical features suggestive of acute parvovirus infection or an unexplained aplastic episode at enrollment. Demographic data, comorbidities, dialysis characteristics, ESA use, and laboratory parameters (hemoglobin, hematocrit, mean corpuscular volume, inflammatory markers, and albumin) were collected. Between-group and within-cohort comparisons used non-parametric tests, and multivariable logistic and linear regression models were used to adjust for age, sex, and other relevant covariates. **Results:** Parvovirus B19 IgG seropositivity was common in both groups (64.9% of hemodialysis patients vs. 48% of controls; crude odds ratio [OR] 2.00, 95% confidence interval [CI] 1.03–3.88, *p* = 0.043). However, hemodialysis patients were substantially older and more often male; after adjustment for age and sex, dialysis status was no longer independently associated with seropositivity (adjusted OR 1.4, 95% CI 0.8–2.3, *p* = 0.20), and within the hemodialysis cohort seropositivity was not associated with age or sex. Hemodialysis patients exhibited significantly lower hemoglobin and hematocrit and higher inflammatory markers than controls, consistent with ESRD-related anemia. Within the hemodialysis cohort, B19 IgG-positive and IgG-negative patients did not differ in hemoglobin, hematocrit, mean corpuscular volume, C-reactive protein, albumin, or ESA use, and IgG serostatus remained unrelated to hemoglobin in a multivariable model adjusting for age, sex, inflammation, nutrition, dialysis vintage, and ESA use (adjusted β = −0.20 g/dL, 95% CI −0.68 to 0.28, *p* = 0.42). Past Parvovirus B19 exposure was therefore not associated with anemia severity or treatment requirements. **Conclusions:** In this cohort of stable maintenance hemodialysis patients, prior Parvovirus B19 exposure, as indicated by IgG seropositivity, was not associated with increased anemia severity, inflammation, or ESA use, and the higher crude seroprevalence in dialysis patients was attributable to their older age rather than to dialysis itself. Because IgG reflects past exposure only and IgM and viral DNA were not assessed, these findings apply strictly to past (IgG-defined) exposure and cannot address active or persistent B19V infection. They suggest that routine Parvovirus B19 IgG screening in asymptomatic hemodialysis patients is unlikely to be useful for anemia management, whereas active or persistent infection—detectable only by molecular testing—remains the more plausible contributor to unexplained or refractory anemia and merits study in selected patients.

## 1. Introduction

Parvovirus B19 (B19V) is a small, non-enveloped, single-stranded DNA virus belonging to the Parvoviridae family that selectively infects human erythroid progenitor cells by binding to the P antigen (globoside) receptor [1,2]. In pediatric populations, B19V is primarily recognized as the causative agent of erythema infectiosum, also known as fifth disease, which typically manifests with a characteristic “slapped cheek” facial rash [3]. While the infection is generally self-limited in healthy, immunocompetent individuals, the virus can induce severe persistent anemia or transient aplastic crises in patients with underlying hematologic conditions or compromised immune systems [4,5,6,7,8]. Patients with end-stage renal disease (ESRD) on maintenance hemodialysis represent a vulnerable population characterized by chronic systemic inflammation, immune dysfunction, and multifactorial anemia [9]. It is increasingly hypothesized that occult viral infections, including B19V, may be a significant yet under-recognized contributor to refractory anemia and erythropoiesis-stimulating agent (ESA) resistance in this cohort. Clinical evidence suggests that B19V can manifest as pure red cell aplasia (PRCA) or severe ESA-resistant anemia, particularly in the context of renal transplantation or during periods of intensified immunosuppression [6,7,8]. However, the precise seroprevalence of B19V and its specific clinical burden in the chronic dialysis population remain insufficiently characterized compared to transplant recipients [9].

In this study, we aimed to determine the seroprevalence of past Parvovirus B19 exposure (IgG positivity) in maintenance hemodialysis patients compared with healthy controls and, more specifically, to test whether IgG-defined prior exposure is associated with persistent differences in erythropoietic parameters—such as lower hemoglobin, altered erythrocyte indices, or greater ESA requirements. We deliberately framed the question around past exposure rather than acute infection: none of our patients had clinical features suggestive of recent parvovirus infection (e.g., a slapped-cheek exanthem, an acute febrile viral illness, or an abrupt unexplained drop in hemoglobin consistent with transient aplastic crisis), and none had a documented episode of unexplained profound anemia. Our hypothesis was that the cumulative healthcare exposure of dialysis patients yields higher rates of IgG-defined prior B19V exposure and that such prior exposure, if it leaves a durable effect on erythropoiesis, would be associated with poorer hematologic outcomes. By testing this hypothesis we sought to clarify whether a history of B19V exposure contributes to ESRD-related anemia and thereby to inform the potential utility of serologic screening. We also aimed to address a gap in the literature, as robust data on B19V in non-transplant dialysis patients remain limited.

## 2. Materials and Methods

### 2.1. Study Design and Participants

This study was designed as a single-center, observational cross-sectional analysis. We included a total of 181 adult participants. The dialysis group consisted of 131 patients with ESRD receiving maintenance hemodialysis at our institution. Inclusion criteria for the dialysis group were age ≥18 years, ESRD treated with maintenance hemodialysis for at least 3 months, and ability to provide informed consent. Patients with clinical or laboratory features suggestive of acute viral infection at the time of sampling were not enrolled. The control group comprised 50 healthy adults without known kidney disease, recruited from hospital staff and patient companions, with no acute illness at sampling, and was intended to reflect background community seroprevalence. The control group was a convenience sample and was not individually matched to the dialysis patients for age or sex; because Parvovirus B19 IgG seroprevalence increases with age, this imbalance was handled analytically by age- and sex-adjusted logistic regression and is also discussed as a limitation (see Section 2.5 and Section 4). Baseline characteristics of both groups (age, sex, comorbidities) were recorded from clinical records and presented for comparison.

### 2.2. Ethics Statement

The study protocol was approved by the Ankara Training and Research Hospital Ethics Committee (Approval No. 2026-02-25; E-25-762). Written informed consent was obtained from all participants prior to enrollment in accordance with the Declaration of Helsinki.

### 2.3. Parvovirus B19 Serological Testing

We used an enzyme-linked immunosorbent assay (ELISA) to detect Parvovirus B19 IgG antibodies in serum (Kit: Parvovirus B19 IgG ELISA, NovaTec Immundiagnostica, Dietzenbach, Germany, with reported sensitivity and specificity >98%). According to the kit instructions, an IgG index value above the established cutoff was considered positive, indicating past exposure or immunity to Parvovirus B19. Equivocal results (grey zone) were excluded from the final analysis (3 participants), whereas a value below the cutoff was considered negative. We deliberately restricted serological testing to IgG because the question addressed in this study concerned the durable, long-term consequences of past, IgG-defined Parvovirus B19 exposure on bone-marrow erythropoietic capacity, rather than the detection of acute or ongoing infection. This design choice was clinically grounded: no participant presented with features suggestive of recent or active Parvovirus B19 infection (such as a slapped-cheek exanthem, an acute febrile viral illness, or an abrupt, unexplained fall in hemoglobin consistent with transient aplastic crisis), and none had a documented history of unexpected profound or transfusion-dependent anemia of the type characteristically associated with acute B19V suppression of erythropoiesis. Accordingly, IgM antibodies and viral DNA were not assessed, and active or persistent infection could not be identified; we treat IgG strictly as a marker of past exposure and address the absence of IgM and molecular testing as a limitation.

### 2.4. Clinical and Laboratory Data

For the dialysis patients, we collected additional clinical data including duration of hemodialysis, frequency of dialysis sessions per week, type of vascular access (arteriovenous fistula or central catheter), use of erythropoiesis-stimulating agents (ESA, e.g., erythropoietin), and presence of comorbid conditions (hypertension, diabetes mellitus, chronic obstructive pulmonary disease (COPD), heart failure, atherosclerotic cardiovascular disease, and any immunosuppressive disease or therapy). We recorded each patient’s routine laboratory results closest to the time of Parvovirus testing, particularly focusing on complete blood count (hemoglobin [HGB], hematocrit [HCT], mean corpuscular volume [MCV], white blood cell count [WBC], platelet count [PLT]) and markers of inflammation/nutrition such as C-reactive protein (CRP) and albumin. These parameters were chosen to assess anemia (HGB/HCT, MCV, and need for ESA) and general health status differences between groups.

### 2.5. Statistical Analysis

All statistical analyses were performed using IBM SPSS Statistics version 26 and R version 4.3. The normality of distribution for continuous variables was assessed using the Kolmogorov–Smirnov test. As most continuous variables were not normally distributed, data are presented as medians with interquartile ranges (IQR). Comparisons between groups were conducted using the Mann–Whitney U test for continuous variables, while categorical variables are expressed as numbers and percentages and were compared using the Chi-square test or Fisher’s exact test, as appropriate. Spearman’s rank correlation was used to evaluate the association between the semiquantitative Parvovirus B19 IgG index and key hematologic parameters within the dialysis cohort. To address the lack of individual age- and sex-matching between the dialysis and control groups, the between-group comparison of seroprevalence was repeated using a multivariable logistic regression model adjusting for age and sex, and the association is reported as an adjusted odds ratio (OR) with a 95% confidence interval (CI). Within the dialysis cohort, a logistic regression model was used to test whether seropositivity was associated with age and sex, and a multivariable linear regression model was used to assess the independent association between IgG serostatus and hemoglobin after adjustment for age, sex, C-reactive protein, albumin, dialysis vintage, and erythropoiesis-stimulating agent (ESA) use; results are reported as regression coefficients (β) with 95% CIs. A separate logistic model examined the association between serostatus and ESA use. Multivariable models used complete-case analysis; the final hemoglobin model included 113 patients owing to missing covariate data. A two-sided *p* value of <0.05 was considered statistically significant.

Artificial intelligence–assisted language tools were used only to improve the clarity, grammar, linguistic fluency, and academic style of the manuscript. AI was not used to generate the study hypothesis, design the methodology, collect data, perform statistical analyses, interpret the results, or draw scientific conclusions. All AI-assisted edits were critically reviewed, verified, and approved by the authors, who take full responsibility for the accuracy, integrity, and final content of the manuscript.

## 3. Results

### 3.1. Study Population and Baseline Characteristics

A total of 181 participants were included in the study, comprising a control group (n = 50) and a hemodialysis group (n = 131). Baseline demographic and clinical characteristics of both groups are summarized in Table 1.

The control group consisted predominantly of female participants (38 women, 76%), whereas males accounted for 12 individuals (24%). In contrast, the hemodialysis group included 71 males (54.2%) and 60 females (45.8%). The two groups therefore differed significantly in sex distribution (χ^2^ test, *p* < 0.001), with a markedly higher proportion of men in the dialysis cohort. Median age also differed significantly between the groups: the control group had a median age of 36.5 years (IQR 27–50), whereas the hemodialysis group had a median age of 61 years (IQR 49–70) (*p* < 0.001). Because both age and sex differed between the groups and Parvovirus B19 seroprevalence is known to increase with age, these imbalances were subsequently addressed by age- and sex-adjusted analysis (Section 3.4).

**Figure 1:**
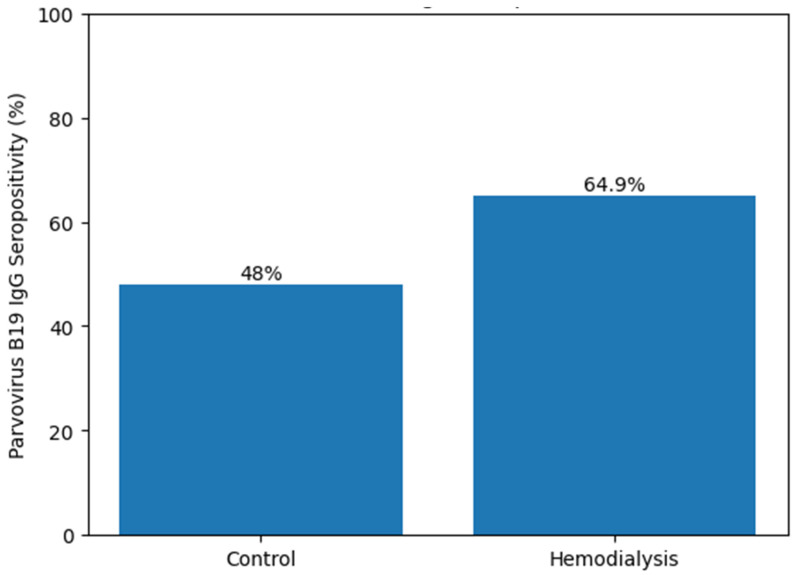
Comparison of Parvovirus B19 IgG seroprevalence between healthy controls and maintenance hemodialysis patients.

Regarding Parvovirus B19 IgG serostatus, 24 individuals (48%) in the control group were seropositive and 26 (52%) were seronegative (Figure 1). In the hemodialysis group, 85 patients (64.9%) were seropositive and 46 (35.1%) were seronegative. In unadjusted analysis, IgG seroprevalence was modestly higher in the hemodialysis group than in controls (64.9% vs. 48%; crude OR 2.00, 95% CI 1.03–3.88, Fisher’s exact *p* = 0.043). However, because the dialysis group was substantially older and more often male, this crude comparison is confounded by age and sex; the age- and sex-adjusted analysis presented in Section 3.4 shows that this difference is no longer significant after accounting for these factors.

### 3.2. Comparison Between Control and Hemodialysis Groups

**Figure 2:**
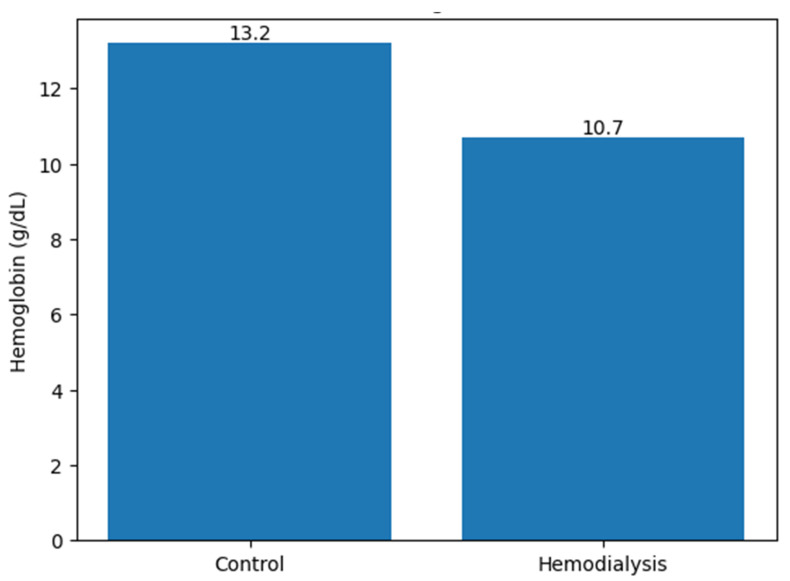
Median hemoglobin levels in control and hemodialysis groups.

Laboratory and clinical parameters of the control and hemodialysis groups are presented in Table 2. Median hemoglobin (HGB) levels were significantly lower in the hemodialysis group compared with controls (10.7 g/dL [IQR 9.60–11.30] vs. 13.2 g/dL [IQR 12.50–14.25], *p* < 0.001) (Figure 2). Similarly, median hematocrit (HCT) values were significantly reduced in hemodialysis patients (33.4% [IQR 29.05–35.7]) compared with controls (39.8% [IQR 37.3–42.2], *p* < 0.001). Median mean corpuscular volume (MCV) was slightly higher in the hemodialysis group (90 fL [IQR 86–93]) than in the control group (88 fL [IQR 84.60–91.35]), and this difference reached statistical significance (*p* = 0.027). Platelet counts were significantly lower in hemodialysis patients than in controls (211 × 10^3^/µL [IQR 167–267] vs. 275 × 10^3^/µL [IQR 246.5–326.5], *p* < 0.001). In contrast, white blood cell (WBC) counts did not differ significantly between groups (7180 cells/µL [IQR 5500–8210] vs. 6870 cells/µL [IQR 6037.5–9660], *p* = 0.528). Inflammatory and nutritional markers also differed between groups. Median C-reactive protein (CRP) levels were significantly higher in the hemodialysis group (6.80 mg/L [IQR 2.4–13]) compared with controls (2.75 mg/L [IQR 1.47–5.07], *p* < 0.001). Serum albumin levels were significantly lower in hemodialysis patients (3.9 g/dL [IQR 3.7–4.2]) than in controls (4.7 g/dL [IQR 4.25–4.90], *p* < 0.001).

### 3.3. Subgroup Analysis Within the Hemodialysis Cohort

Subgroup analyses were performed within the hemodialysis cohort according to Parvovirus B19 serostatus (Table 3). Sex distribution did not differ significantly between Parvovirus IgG–seropositive and seronegative patients (54.1% vs. 54.3% male, *p* = 0.980). Median hemoglobin levels were identical in both subgroups (10.7 g/dL; seronegative IQR 10.1–11.3 g/dL vs. seropositive IQR 9.5–11.4 g/dL; *p* = 0.659). No significant differences were observed in hematocrit (*p* = 0.652) or mean corpuscular volume (*p* = 0.380). Median platelet counts were similar between seronegative (204.5 × 10^3^/µL [IQR 165–265]) and seropositive patients (217 × 10^3^/µL [IQR 171–265]) (*p* = 0.581). Median CRP levels also did not differ significantly between the two subgroups (7.2 mg/L [IQR 3.1–12.6] vs. 6.7 mg/L [IQR 2.4–11.6], *p* = 0.862). Serum albumin levels were comparable in both groups (3.9 vs. 3.9 g/dL, *p* = 0.749). Clinical treatment characteristics were similarly distributed. Median duration of hemodialysis was 4 years in both subgroups (*p* = 0.629), and weekly dialysis frequency was identical (*p* = 0.989). The proportion of patients receiving erythropoiesis-stimulating agents was similar between seronegative (69.6%) and seropositive patients (67.1%) (*p* = 0.769). Vascular access type did not differ significantly by serostatus: arteriovenous fistula was used in 65.2% of seronegative and 71.8% of seropositive patients (*p* = 0.437), while catheter use was observed in 30.4% and 27.1% of patients, respectively (*p* = 0.682).

The prevalence of comorbid conditions, including hypertension, diabetes mellitus, chronic obstructive pulmonary disease, heart failure, immunosuppressive diseases, and atherosclerotic cardiovascular disease, was comparable between Parvovirus-seronegative and seropositive patients (all *p* > 0.05).

### 3.4. Age- and Sex-Adjusted and Multivariable Analyses

Because the dialysis and control groups differed significantly in age and sex, and because Parvovirus B19 IgG seroprevalence increases with age, the crude between-group comparison was repeated in a logistic regression model adjusting for age and sex. After adjustment, dialysis status was no longer independently associated with seropositivity (adjusted OR 1.4, 95% CI 0.8–2.3, *p* = 0.20), indicating that the higher crude seroprevalence observed in the dialysis group (crude OR 2.00, 95% CI 1.03–3.88, *p* = 0.043) was largely attributable to the older age and different sex distribution of the dialysis cohort rather than to dialysis status per se. Consistent with this, within the hemodialysis cohort seropositivity was not associated with age (OR per year 1.00, 95% CI 0.98–1.03, *p* = 0.73) or male sex (OR 0.98, 95% CI 0.48–2.02, *p* = 0.95) in a logistic regression model, and these associations remained non-significant after additional adjustment for dialysis vintage and transfusion history.

To determine whether past Parvovirus B19 exposure was independently associated with anemia severity, a multivariable linear regression model was constructed with hemoglobin as the dependent variable and IgG serostatus, age, sex, C-reactive protein, serum albumin, dialysis vintage, and ESA use as covariates (n = 113; adjusted R^2^ = 0.19). IgG seropositivity was not independently associated with hemoglobin (adjusted β = −0.20 g/dL, 95% CI −0.68 to 0.28, *p* = 0.42). In the same model, higher CRP (*p* < 0.05) and ESA use (*p* < 0.05) were associated with lower hemoglobin, whereas higher serum albumin and older age were associated with higher hemoglobin, consistent with the established determinants of anemia in this population. A separate logistic regression model showed no association between IgG serostatus and ESA use (adjusted OR 0.88, 95% CI 0.39–2.00, *p* = 0.76).

Finally, to assess a possible dose-dependent relationship, the semiquantitative Parvovirus B19 IgG index was correlated with hemoglobin using Spearman’s rank correlation. No significant correlation was found across the full cohort (ρ = −0.09, *p* = 0.29) or among seropositive patients only (ρ = −0.13, *p* = 0.24), providing no evidence of a dose–response association between antibody level and anemia severity. Taken together, these adjusted and multivariable analyses indicate that past Parvovirus B19 exposure is not independently associated with anemia severity, erythrocyte indices, inflammation, or ESA requirements in maintenance hemodialysis patients.

## 4. Discussion

In this study, we investigated whether prior infection with human Parvovirus B19 might contribute to anemia in patients undergoing chronic hemodialysis. This question was motivated by the well-established tropism of Parvovirus B19 for erythroid precursor cells and its capacity to cause anemia, particularly in immunocompromised hosts, as well as by the clinical observation that anemia in end-stage renal disease (ESRD) can occasionally be refractory to standard therapeutic approaches. To address this, we compared the seroprevalence of Parvovirus B19 between dialysis patients and healthy controls and examined whether hematologic or clinical parameters differed within the dialysis cohort according to Parvovirus serostatus.

We found that Parvovirus B19 IgG seropositivity was common in both healthy individuals and dialysis patients, with rates of approximately 50–65% in our sample. Although the crude prevalence was higher in the dialysis group than in controls (65% vs. 48%; crude OR 2.00, *p* = 0.043), this difference was no longer significant after adjustment for age and sex (adjusted OR 1.4, *p* = 0.20) and was therefore attributable to the older age of the dialysis cohort rather than to dialysis itself. Furthermore, within the dialysis cohort, there was no evidence that patients with IgG-defined prior Parvovirus B19 exposure were more anemic or had higher inflammatory markers than those who were IgG-negative. Hemoglobin levels, hematocrit, mean corpuscular volume (MCV), C-reactive protein (CRP), serum albumin, and the use of erythropoiesis-stimulating agents (ESAs) were all statistically comparable between Parvovirus-positive and -negative patients, and IgG serostatus was not independently associated with hemoglobin in multivariable analysis. Taken together, these findings do not support the hypothesis that IgG-defined prior Parvovirus B19 exposure is a major contributor to anemia or ESA resistance in the hemodialysis population.

Our results should be interpreted within an appropriate epidemiological context. In unadjusted analysis, IgG seroprevalence was modestly higher in dialysis patients than in controls (64.9% vs. 48%; crude OR 2.00, 95% CI 1.03–3.88, *p* = 0.043). However, dialysis patients were substantially older and more frequently male, and Parvovirus B19 seroprevalence is known to increase with age. After adjustment for age and sex in a logistic regression model, dialysis status was no longer independently associated with seropositivity (adjusted OR 1.4, 95% CI 0.8–2.3, *p* = 0.20). This indicates that the higher crude seroprevalence in dialysis patients was largely explained by their older age and different sex distribution rather than by dialysis itself or by greater cumulative healthcare exposure. Consistent with this, within the dialysis cohort seropositivity was not associated with age or sex. If repeated healthcare exposures—including historical blood transfusions and frequent hospital contact—do increase Parvovirus B19 exposure, the effect appears modest, or our study may have been underpowered to detect it. Our findings are consistent with the study by Alves et al., who similarly reported no significant association between B19V DNA positivity and anemia, duration of dialysis, blood transfusion history, renal transplantation, age, or sex [10].

Notably, previous studies from different geographic regions have reported variable Parvovirus B19 seroprevalence among patients with chronic kidney disease. The overall IgG seroprevalence observed in our study (~65% in dialysis patients and ~50% in controls) falls within the range commonly reported for adult populations, which typically varies between 40% and 60% depending on age and geographic location [11,12,13]. This further supports the interpretation that our dialysis cohort did not have an unusually high exposure history compared with the general population.

We did not identify any association between Parvovirus B19 serostatus and anemia severity among dialysis patients. These findings indicate that prior infection, as reflected by IgG positivity, is unlikely to be a determinant of current hemoglobin levels or responsiveness to ESA therapy. A plausible explanation is that any clinically meaningful effect of Parvovirus B19 on erythropoiesis would be mediated by active or persistent infection rather than by IgG-defined prior exposure. Because our study did not assess acute infection or viremia (e.g., IgM serology or PCR), our analysis primarily reflects exposure history. In individuals with prior exposure, the presence of IgG antibodies indicates immune memory rather than ongoing viral suppression of red cell production, making it biologically reasonable that their anemia profile would resemble that of never-infected individuals.

In contrast, persistent Parvovirus B19 infection has been well documented as a cause of significant anemia, particularly pure red cell aplasia, in immunocompromised hosts, including dialysis and especially kidney transplant patients. A recent multicenter study from Brazil reported an unexpectedly high prevalence of persistent Parvovirus B19 DNA viremia in asymptomatic dialysis patients (65% vs. 6.3% in healthy controls), although overt clinical manifestations were not observed in that cohort [11]. Conversely, a study from Egypt demonstrated that active Parvovirus B19 infection detected by PCR was significantly more common in hemodialysis patients than in controls and that viremic patients had significantly lower hemoglobin levels and red blood cell indices [13]. Together, these studies highlight that active Parvovirus B19 infection can occur in the dialysis population and may contribute to anemia under certain circumstances.

Our findings do not contradict these reports but rather emphasize an important distinction: serologic evidence of past exposure does not necessarily reflect current clinically relevant infection. IgG positivity denotes prior exposure, whereas active viral replication, which is more likely to influence erythropoiesis, can be detected only through molecular methods. In our cohort, the absence of an association between IgG status and anemia may reflect the limited ability of serology alone to identify ongoing infection, particularly in immunocompromised patients who may exhibit atypical or blunted antibody responses.

From a clinical perspective, our results suggest that routine screening for Parvovirus B19 IgG in dialysis patients is unlikely to be informative for anemia management, as serostatus was not associated with anemia severity or ESA use. Anemia in dialysis patients is predominantly attributable to ESRD-related mechanisms, including inadequate erythropoietin production and chronic inflammation, and our data indicate that a history of Parvovirus B19 infection does not independently exacerbate this condition. Nevertheless, clinicians should remain aware that active Parvovirus B19 infection, although probably uncommon, may represent an underrecognized cause of unexplained or ESA-resistant anemia in selected patients. In such cases, particularly among immunosuppressed individuals or those being evaluated for kidney transplantation, testing for Parvovirus B19 DNA may be warranted. Overall, our study contributes to the existing literature by demonstrating that, in a stable outpatient hemodialysis population, IgG seropositivity alone is not clinically detrimental; rather, it is the presence of ongoing viral replication, if any, that is likely to be of clinical relevance.

### Limitations of the Study

We acknowledge several limitations. First, the sample size, particularly of the control group, was relatively modest, which may limit power to detect small differences. Second, the control group was a convenience sample that was not individually matched to the dialysis patients and was younger and predominantly female; because Parvovirus B19 IgG seroprevalence increases with age, we addressed this imbalance using age- and sex-adjusted logistic regression, which showed that dialysis status was not independently associated with seropositivity. Nonetheless, residual confounding cannot be excluded, and individual-level control data were not available for all analyses. Third, and most importantly, we assessed only Parvovirus B19 IgG. We did not test for IgM or viral DNA, so active or persistent infection could not be identified; our findings therefore apply strictly to past (IgG-defined) exposure and cannot exclude a contribution of subclinical viremia to anemia in a small number of patients. Fourth, several determinants of renal anemia and of ESA responsiveness were not available in our dataset and could not be incorporated: iron status (ferritin, transferrin saturation) and iron therapy, reticulocyte count, transfusion burden beyond a binary history, dialysis adequacy (Kt/V), and detailed ESA dosing or a formal ESA resistance index. ESA exposure was captured only as a binary variable, precluding calculation of weight-adjusted ESA dose or an erythropoietin ESA resistance index; residual confounding by these factors is therefore possible, and our anemia analyses should be interpreted with this in mind. Fifth, the study was cross-sectional, measuring exposure and anemia parameters at a single time point, which precludes inferences about causality or temporality. Finally, this was a single-center study in one geographic region, which may limit generalizability, as Parvovirus B19 seroprevalence and anemia-management practices vary across regions and centers.

## 5. Conclusions

In conclusion, although hemodialysis patients had a high prevalence of past Parvovirus B19 exposure (IgG positivity), this prevalence was only modestly higher than in younger controls and was no longer significant after adjustment for age and sex, indicating that the difference reflected the older age of dialysis patients rather than dialysis itself. Importantly, past Parvovirus B19 exposure was not associated with the degree of anemia, erythrocyte indices, inflammation, or ESA requirements, and IgG serostatus was not independently associated with hemoglobin in multivariable analysis. Because only IgG was assessed, these conclusions apply strictly to past (IgG-defined) exposure; they suggest that routine Parvovirus B19 IgG screening in asymptomatic dialysis patients is unlikely to be useful for anemia management. They do not exclude a role for active or persistent infection, which can be identified only by molecular testing (PCR for viremia) and remains the more plausible contributor to unexplained or ESA-refractory anemia in selected patients. Clinicians should continue to address the well-established causes of anemia in dialysis patients and consider active Parvovirus B19 infection in cases of unexplained refractory anemia on an individual basis. Future research should focus on identifying active B19V infection in ESRD patients, ideally through longitudinal and molecular studies, to determine its clinical significance.

## Figures and Tables

**Table 1:** Demographic data of the control and dialysis groups.

Variables	Control Group (n = 50)	Dialysis Group (n = 131)	*p* Value
Age, years	36.5 (27–50)	61 (49–70)	<0.001 ^a^
Gender, n (%)			
Male	12 (24)	71 (54.2)	<0.001 ^b^
Female	38 (76)	60 (45.8)	
Parvovirus serostatus, n (%)			
Positive	24 (48)	85 (64.9)	
Negative	26 (52)	46 (35.1)	0.043 ^c^

Mann–Whitney U test ^a^, Pearson Chi-square test ^b^, Fisher’s exact test ^c^. Continuous variables are expressed as median (interquartile range); categorical variables as numbers and percentages.

**Table 2:** Comparison of laboratory and clinical data.

Variables	Control Group (n = 50)	Dialysis Group (n = 131)	*p* Value
HGB, g/dL	13.2 (12.50–14.25)	10.7 (9.60–11.30)	<0.001 ^a^
WBC, cells/µL	6870 (6037.5–9660.00)	7180 (5500–8210)	0.528 ^a^
HCT, (%)	39.8 (37.3–42.2)	33.4 (29.05–35.7)	<0.001 ^a^
MCV, fL	88 (84.60–91.35)	90 (86–93)	0.027 ^a^
PLT, ×10^3^/µL	275 (246.5–326.5)	211 (167–267)	<0.001 ^a^
CRP, mg/L	2.75 (1.47–5.07)	6.80 (2.4–13)	<0.001 ^a^
Albumin, g/dL	4.7 (4.25–4.90)	3.9 (3.7–4.2)	<0.001 ^a^

Mann–Whitney U test ^a^. Continuous variables are expressed as median (interquartile range). Abbreviations: HGB: Hemoglobin, WBC: White blood cell, HCT: Hematocrit, MCV: Mean Corpuscular Volume, PLT: platelet, CRP: C-reactive protein, µL: microliter, fL: femtoliter.

**Table 3:** Subgroup Analysis of Dialysis Patients.

Variables	Parvovirus Seronegative (n = 46)	Parvovirus Seropositive (n = 85)	*p* Value
Age, years	60 (45.5–69.5)	61 (50–70)	0.862 ^b^
Gender			
Male Female	25 (54.3) 21 (45.7)	46 (54.1) 39 (45.9)	0.980 ^a^
HGB, g/dL	10.7 (10.1–11.3)	10.7 (9.5–11.4)	0.659 ^b^
HCT, (%)	33.2 (31.4–35.8)	33.8 (29.1–35.6)	0.652 ^b^
MCV, fL	90 (87–94.5)	90 (85–92)	0.380 ^b^
PLT, ×10^3^/µL	204.5 (165–265)	217 (171–265)	0.581 ^b^
CRP, mg/L	7.2 (3.1–12.6)	6.7 (2.4–11.6)	0.862 ^b^
Albumin, g/dL	3.9 (3.7–4.2)	3.9 (3.7–4.2)	0.749 ^b^
Duration of Hemodialysis, years	4 (2–7)	4 (2–9)	0.629 ^b^
Hemodialysis Frequency, per week	3 (3–3)	3 (3–3)	0.989 ^b^
Hypertension	33 (71.7)	70 (82.4)	0.157 ^a^
Diabetes Mellitus	17 (37)	31 (36.5)	0.956 ^a^
COPD	4 (8.7)	4 (4.7)	0.450 ^c^
Atherosclerotic cardiovascular disease	16 (34.8)	22 (25.9)	0.284 ^a^
Heart Failure	11 (23.9)	19 (22.4)	0.839 ^a^
Immunosuppressive disease	0 (0)	1 (1.2)	1.000 ^c^
Erythropoiesis-stimulating agent use	32 (69.6)	57 (67.1)	0.769 ^a^
Vascular Access			
Catheter	14 (30.4)	23 (27.1)	0.682 ^a^
Arteriovenous Fistula	30 (65.2)	61 (71.8)	0.437 ^a^

Pearson Chi-square test ^a^, Mann–Whitney U test ^b^, Fisher’s exact test ^c^. Continuous variables are expressed as median (interquartile range); categorical variables as numbers and percentages. Vascular access data were available for 44 seronegative and 84 seropositive patients. Abbreviations: HGB: Hemoglobin, HCT: Hematocrit, MCV: Mean Corpuscular Volume, PLT: platelet, CRP: C-reactive protein, COPD: Chronic Obstructive Pulmonary Disease, µL: microliter, fL: femtoliter.

## Data Availability

The datasets generated and/or analyzed during the current study are available from the corresponding author on reasonable request.
